# Effectiveness of Chemical Sanitizers against *Salmonella* Typhimurium in Nutrient Film Technique (NFT) Hydroponic Systems: Implications for Food Safety, Crop Quality, and Nutrient Content in Leafy Greens

**DOI:** 10.3390/foods13121929

**Published:** 2024-06-19

**Authors:** Abigail A. Mensah, Melanie L. Lewis Ivey, Margaret R. Moodispaw, Sanja Ilic

**Affiliations:** 1 Human Nutrition, Department of Human Sciences, College of Education and Human Ecology, The Ohio State University, Columbus, OH 43210, USA; mensah.100@osu.edu; 2 Department of Plant Pathology, College of Food, Agricultural and Environmental Sciences, The Ohio State University, Wooster, OH 44691, USA; 3 North Central Regional Plant Introduction Station, United States Department of Agriculture-Agriculture Research Service (USDA-ARS), Ames, IA 50011, USA; margaret.moodispaw@usda.gov

**Keywords:** sanitizer, nutrient solution, nutrient film technique, food safety, hydroponics, *Salmonella* Typhimurium, plant health and quality, pathogen elimination, SaniDate 12.0, nutritional value

## Abstract

Hydroponic farming systems play an increasingly important role in the sustainable production of nutrient-rich foods. The contamination of surfaces in hydroponic fresh produce production poses risks to the food safety of crops, potentially endangering public health and causing economic losses in the industry. While sanitizers are widely used in commercial hydroponic farms, their effectiveness against human pathogens on surfaces and their impact on plant health and quality are not known. In this study, we evaluated the efficacy of chemical sanitizers in eliminating *Salmonella* Typhimurium from inanimate surfaces in commercial hydroponic Nutrient Film Technique (NFT) systems. Further, we assessed the impact of sanitizers on the yield, quality, and nutritional value of lettuce and basil. Sanitizers (Virkon, LanXess, Pittsburgh, PA, USA; SaniDate 12.0, BioSafe Systems, East Hartford, CT, USA; KleenGrow, Pace Chemical Ltd., Delta, BC, Canada; Green Shield, United Labs Inc., St Charles, IL, USA; Zerotol, BioSafe Systems, East Hartford, CT, USA; Bleach, Pure Bright, ON, Canada) were tested against *Salmonella* Typhimurium inoculated on NFT surfaces (nutrient reservoir, growing channels, top covers, drain lines). The effective treatments were then tested for their impact on lettuce and basil in a split-plot experiment conducted in commercial NFT units. Crop yield, color, and nutrient content (chlorophyll and carotenoids) were measured throughout the crop life cycle. While all quaternary ammonium compounds (QAC), SaniDate 12.0 (200 ppm), Zorotol (5%), and Virkon (1%) eliminated *Salmonella* Typhimurium from commercial NFT surfaces, chlorine-based sanitizer treatments were statistically similar to water treatments on most surfaces. All chemical sanitizers impacted the yield, color, and nutritional value of lettuce and basil. SaniDate 12.0 (200 ppm) was the least detrimental to crops and was identified as a potential candidate for further validation in commercial hydroponic settings. The findings of this study will be translated into recommendations for the industry and will contribute to the development of future food safety guidelines and policies.

## 1. Introduction

The safety of food and its nutritional value play a critical role in achieving ongoing public health goals aimed at reducing diseases linked to diets [1]. Fresh produce contamination and associated foodborne disease outbreaks rank as top priorities in public health concerns in the United States (US) and globally [2,3]. Fresh produce, such as leafy greens, are increasingly produced in hydroponic systems to meet the rising demand for nutrient-dense foods. While hydroponic production in controlled environments is generally perceived as safer than open-field cultivation, outbreaks of foodborne illnesses caused by Salmonella spp. and STEC associated with hydroponically grown produce have been reported [4,5,6]. Contaminated water sources, nutrient solutions, and inadequate sanitation practices within hydroponic systems have been identified as high-risk food safety hazards and recommended to be prioritized for food safety management [7].

Hydroponic systems present unique challenges when it comes to sanitation, as the recirculating water and nutrient solutions can potentially harbor and facilitate the growth of microbial pathogens. While chemical sanitizers like sodium hypochlorite, peracetic acid (PAA), and quaternary ammonium compounds (QACs) are commonly used to eliminate human pathogens on food contact surfaces and in produce washes [8,9,10], their effectiveness in hydroponic systems has not been adequately validated and may have a detrimental effect on plant health. This is especially true for leafy greens because of their delicate structure [11,12,13].

The health aspects of fresh leafy greens are major drivers for their consumption [14]. Lettuce and basil are excellent sources of vitamins and various phytochemicals [15,16]. For instance, carotenoids, which are abundant in dark leafy greens, safeguard human cells and tissues from oxidative damage caused by free radicals, thus reducing the risk of cancer, heart disease, and age-related eye disorders [17,18,19]. They also play an important role in skin health and immunity [20,21]. Chlorophyll is the primary pigment in leafy greens that enables plants to grow and survive through the process of photosynthesis [17]. In addition to its potential anti-carcinogenic and antimutagenic activities, chlorophyll determines the crop organoleptic quality and affects the marketability of the product [17]. The introduction of any mitigation strategies during production may impact the concentrations of these valuable natural compounds, reduce nutritional quality, and diminish their marketable yield. 

Validating and establishing best practices for sanitation in hydroponic systems are necessary to ensure the safety and nutritional integrity of hydroponically grown produce. These efforts can help bridge the gap in recommendations for mitigating food safety hazards in hydroponics while producing high-quality, nutrient-dense produce. Studies tailored to commercial hydroponic systems, specifically nutrient film technology (NFT), were conducted to (1) evaluate the effectiveness of sanitizers in eliminating *Salmonella* Typhimurium on inanimate NFT surfaces and (2) assess the effect of sanitizers on lettuce and basil yield, quality, and nutritional value.

## 2. Materials and Methods

### 2.1. Effectiveness of Chemical Sanitizers against Salmonella Typhimurium on NFT Surfaces

#### 2.1.1. Experimental Design and Hydroponic Surfaces 

Surfaces specific to CropKing^®^ NFT systems, including the growing channels and top covers (food-grade, UV stabilized PVC), the main drain line (4″ PVC tube), and reservoirs (ABS plastic with UV inhibitors) were tested in this study. The NFT nutrient reservoir was cut into 5 cm × 5 cm (25 cm^2^) coupons, and the growing channels, top covers, and main drain line were cut into 5 cm strips that resulted in 66.0, 70.0, and 62.8 cm^2^ pieces, respectively. The experiment was conducted twice, and each experiment was arranged in a complete block design with four replicates (*n* = 4). Standard BSL-2 laboratory procedures were followed for all experiments as described by The Ohio State University-IBC approved protocol No. 2018R00000006.

#### 2.1.2. Bacterial Strain and Inoculation of Surfaces

*Salmonella* spp. are commonly linked to outbreaks and recalls of hydroponic crops. Therefore, we chose *Salmonella enterica* Typhimurium LT2 (strain JSG626 responsible for gastroenteritis in humans) [22] modified for resistance to 75 µG/mL kanamycin [23] was grown in 10 mL of Luria-Bertani broth (LB; Thermo Fisher Scientific, Waltham, MA, USA), for 18 h at 35 °C with agitation (120 rpm). The liquid culture was then centrifuged (4122× *g*; 5 min) (Sorvall Legend XTR, Thermo Fisher Scientific, Langenselbold, Germany), the pellet was rinsed twice with 1× phosphate-buffered solution (PBS, pH of 7.4, 0.01 M, Thermo Fisher Scientific, Waltham, MA, USA) then suspended in Hydro-Gro Leafy Greens nutrient solution (1.2 g/L) (CropKing Inc., Lodi, OH, USA) supplemented with calcium nitrate (1 g/L). For each surface type, the inner surface directly exposed to the nutrient solution was inoculated by dispensing 5 × 0.2 mL drops of inoculum directly on the surface, achieving a concentration of ~10^6^ CFU/cm^2^. Depending on the surface type, the inoculated surfaces were dried in a laminar flow hood for up to three hours. 

#### 2.1.3. Sanitizer Application, Sample Processing, and Bacterial Enumeration

The active ingredients, concentration, and contact times of the sanitizers are listed in Table 1. Surface samples were placed in sterile glass cake pan dishes and sprayed with sanitizer until run-off. Inoculated surfaces treated with water were used as controls. Following the prescribed contact time, the reservoir surfaces were transferred to Whirl-Pak bags containing 25 mL of sterile 1X PBS, and the growing channels, top covers, and main drain lines were transferred into Whirl-Pak bags containing 140 mL of sterile 1X PBS. The samples were then sonicated in a 75HT Ultrasonic Cleaner (VWR International, Radnor, PA, USA) at 40 Hz for 2 min to dislodge any remaining cells. For each surface, 10-fold serial dilutions were prepared using 1xPBS and spread plated in duplicate onto xylose lysine deoxycholate (XLD) (HiMedia Laboratories, Mumbai, India) (75 µg/mL kanamycin) agar. The plates were incubated for up to 48 h at 35 °C, and colonies were enumerated. Each sample was enriched in 50–200 mL in a tetrathionate broth base (TBB; Fisher Scientific, Hampton, NH, USA) at 35 °C for 24 h, spread-plated on XLD and the presence or absence of *S*. Typhimurium was determined based on colony morphology after overnight incubation at 35 °C.

### 2.2. Impact of Chemical Sanitizers on Yield and Nutritional Attributes of Lettuce and Basil Grown in NFT Hydroponic System

#### 2.2.1. Experimental Design and Crop Production

**Figure 1:**
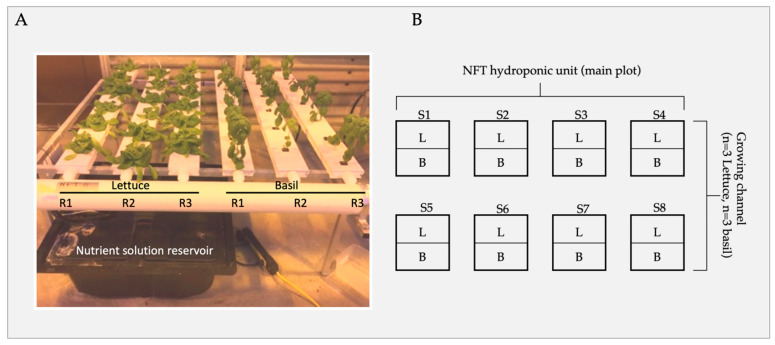
Experimental design. (**A**) Nutrient flow technology (NFT) hydroponic unit (main plot S) consisted of six growing channels: three lettuce (*n* = 3 channels; subplot L) and basil (*n* = 3 channels; subplot B), each containing six plants per channel. (**B**) A schematic of the split-plot experimental set-up. Each plot (S1–8) received different treatments. Crops were randomly sampled weekly until harvest (Day 1, Day 7, Day 14, Day 21, Day 28).

Eight NFT 4–6 systems were set up in a BSL-2 research greenhouse located at The Ohio State University, CFAES-Wooster, Ohio. Greenhouse temperatures were set to 30 °C at daytime and 4 °C at nighttime. Plants were exposed to a 12-h-photoperiod. To model the exposure effect of these sanitizers on leafy greens, a single NFT system was treated with one sanitizer and consisted of six channels with three basil and three lettuce plants per channel. The experiment was a split plot design, with eight main plots (sanitizer treatments and control), split between lettuce and basil, arranged as a completely randomized design (CRD) (Figure 1). The experiment was conducted three times.

Lettuce (*Lactuca sativa* cv. Rex) and basil (*Ocimum basilicum* cv. Genovese) were propagated in Rockwool pads (2.5 cm × 2.5 cm × 3.8 cm) soaked with Hydro-Gro Leafy Greens nutrient solution (1.2 g/L) (CropKing Inc., Lodi, OH) supplemented with calcium nitrate (1 g/L) adjusted to pH 5.5–6 with 1M KOH and 30% HCL. The seedlings were transferred to the NFT 4–6 systems at the 2–4 true-leaf stage (~2 weeks old seedlings) and allowed 5 days to adapt in the system.

#### 2.2.2. Treatment of Nutrient Solution with Sanitizers and Measurement of Fresh Edible Weight (Marketable Yield) and Leaf and Root Color 

Sanitizer was added to the reservoirs (95 L) containing the nutrient solution 5 days post-transplanting to achieve the desired concentration (Table 1). Electrical conductivity (EC) and pH were continuously monitored using in-line Bluelab Guardian Monitor Connect instrumentation (Bluelab Corporation Limited, Tauranga, New Zealand) and adjusted as needed to maintain a pH 5.5–6.0 and EC = 1.4–2.1 mS/cm. For the SaniDate 12.0 and Bleach treatments, peroxyacetic acid (PAA; mg/L), chlorine residuals (mg/L), and the oxidation–reduction potential (ORP; mV), respectively, were measured using two sensors (chlorine higher range (CHR) and chlorine-free high range (FHR, temperature < 20 °C) sensors) at each sampling time using Kemio^TM^ (Palintest, Golden, CO, USA) according to the manufacturer’s instructions. Measurements were stopped when the detected residual dropped below 3%. Three plants (one plant per replicate) were randomly sampled every week (time; T: 1, 7, 14, 21, 28 days), and the Rockwool–root matrix was separated from the edible leaves (shoots) at the Rockwool–stem line. When possible, the roots were split from the Rockwool. The weight of the Rockwool–root matrix, roots, and shoots was recorded, and the color (red-green-blue, RGB) of the leaves and roots was measured using the RGB color detector mobile application (Version 2.5.02). The hexadecimal color value (HEX) containing a six-alphanumeric symbol code made of three/two-symbol elements determining red, blue, and green for each treatment was measured. Leaf sample aliquots were preserved for nutrient composition as described below. 

#### 2.2.3. Impact of Chemical Sanitizers on Nutritional Content (Chlorophyll and Carotenoid) of Lettuce and Basil 

Chlorophyll and carotenoid concentrations were determined using spectrophotometry by measuring the absorbance of the extracts at various wavelengths, according to Lichtenthaler et al. (1987) [24]. Leaves (0.2 g) were suspended in 15 mL 80% acetone and incubated in the dark at ambient room temperature (25 °C) until the leaves were bleached (24 to 48 h). A volume of 1 mL of the supernatant was extracted, and the absorbance was measured at 663.2 nm, 646.8 nm (chlorophyll), and 470 nm (carotenoids) using a 50 Bio UV-Visible Spectrophotometer (Varian, Palo Alto, CA, USA). The concentration of chlorophyll and carotenoids in the leaves was calculated using the formulas described by Lichtenthaler et al. (1987) [24] (Table 2).

### 2.3. Statistical Analysis 

Data were organized and managed in Excel (Microsoft 2010; Redmond, WA, USA), and all statistical tests were performed using STATA (Stata/BE 17.0 (Intel 64-bit)). Interexperimental variability between experiments was tested using Levene’s test of equal variance (*p* < 0.05) [25,26]. Due to the different inoculum drying times used in the two experiments, the data were analyzed and presented independently (Table 3 and Table 4). Cell counts were log-transformed, and the log reduction and percentage reduction were calculated. The differences between sanitizer treatments and the water-treated control were analyzed using the Kruskal–Wallis test, and medians were separated using Dunn’s non-parametric pairwise multiple comparisons [25].

The data from three experiments testing the yield and nutrients in lettuce and basil were pooled in the statistical analysis due to non-significant interexperimental variability (*p* = 0.767). The fresh edible weight, RGB values, chlorophyll, and carotenoid concentrations were compared with water-treated control and analyzed in the GLM (Generalized Linear Model) at α = 0.05, and means were separated using Fisher’s least significant difference test. 

## 3. Results

### 3.1. Effectiveness of Chemical Sanitizers on Eliminating Salmonella Typhimurium from Inanimate NFT Surfaces 

Chlorine-based sanitizers and water treatment achieved variable results depending on the surface type. None of the chlorine-based treatments eliminated (100% reduction and no detection after enrichment) *S.* Typhimurium from any of the surfaces tested. The achieved reduction for chlorine-based treatments in experiment 1 ranged between 69.342% (0.86 ± 0.43 log CFU/cm^2^) to 99.907% (4.14 ± 0.40 log CFU/cm^2^), depending on the surface type (Table 3). Reduction of *S.* Typhimurium with the water treatment ranged from 64.000% (0.45 ± 0.19 log CFU/cm^2^) on the reservoir surface to 99.559% (2.36 ± 0.60 log CFU/cm^2^) on the main drain line. For experiment 2, the achieved reduction for chlorine-based treatments ranged between 99.870% (3.65 ± 0.61 log CFU/cm^2^) to 99.999% (5.34 ± 0.26 log CFU/cm^2^), depending on the surface type (Table 4). Water treatment reduced *S.* Typhimurium from 99.951% (3.68 ± 0.35 log CFU/cm^2^) on the growing channel to 99.997% (4.90 ± 0.16 log CFU/cm^2^) on the main drain line. On the reservoir, the top cover, the growing channel, and the main drain line, water was as effective as chlorine-based treatments in both experiments. Although a 99.999–100% reduction was achieved with 200 ppm bleach (growing channel and the main drain line) and NatriClor CD (reservoir), *S.* Typhimurium was detected after enrichment.

The PAA-based sanitizers Zerotol and SaniDate 12.0 (200 ppm only) were effective in eliminating *S.* Typhimurium from all the surfaces tested in this study compared to the water treatment (Table 3 and Table 4). SaniDate 12.0 (100 ppm) eliminated *S.* Typhimurium from the reservoir (100%; 5.60 ± 0.00 log CFU/cm^2^) and top cover (100%; 5.18 ± 0.00 log CFU/cm^2^) it was not eliminated from the main drain line (99.926%; 4.24 ± 0.51 log CFU/cm^2^), or the growing channel (99.971%; 4.49 ± 0.35 log CFU/cm^2^ reduction) in the first experiment (Table 3). For experiment two, SaniDate 12.0 (100 ppm) eliminated *S.* Typhimurium on reservoir (100%; 5.60 ± 0.00 log CFU/cm^2^), top cover (100%; 5.18 ± 0.00 log CFU/cm^2^) and growing channel (100%; 5.15 ± 0.00 log CFU/cm^2^), but not on the main drain line (99.999%; 5.03 ± 0.10 log CFU/cm^2^) (Table 4).

The quaternary ammonia-based (QAC) (Green Shield, KleenGrow) and PPMS-based (Virkon) sanitizers eliminated (100%; 5.15 ± 0.00 to 5.60 ± 0.00 log CFU/cm^2^) *S.* Typhimurium from all the surfaces (Table 3 and Table 4).

### 3.2. Effect of Chemical Sanitizers on Yield, Quality, and Nutritional Value of Lettuce and Basil Grown in NFT Hydroponic Systems

#### 3.2.1. Sanitizer Residues 

Following treatment of the nutrient solution with bleach (100 ppm), free chlorine was detected until Day 7 (0.15 ± 0.29 mg/L), while total chlorine dissipated within seven days (Table 5). For the 200 ppm bleach, free chlorine (0.81 ± 1.69 mg/L) and total chlorine (1.25 ± 1.81 mg/L) were detected 14 days post-treatment, and the concentrations were comparable to the non-treated nutrient solution. The initial oxidation redox ORP for bleach (100 ppm and 200 ppm) was 872.33 ± 33.45 mV and 857.67 ± 25.45 mv. The bleach declined by 94.6% for 100 ppm bleach and 60.58% for 200 ppm bleach by Day 14.

For the 200 ppm SaniDate 12.0 solution, the active ingredient, peroxyacetic acid (PAA), dissipated to less than 5 mg/L within the first 24 h. The QAC and PPMS-based sanitizers were not measured due to the plants dying within the first 5 days.

#### 3.2.2. Quality and Yield

The color of basil and lettuce leaves grown in nutrient solutions treated with bleach (100 ppm and 200 ppm) and SaniDate 12.0 (200 ppm) changed by harvest (Day 28), resulting in different HEX codes compared to the non-treated control (Table 6). For lettuce, there were no significant differences in the R, G, and B values (*p* = 0.901 [R], *p* = 0.730 [G], *p* = 0.629 [B]) between the sanitizers and the control. For basil, the R and G values (*p* = 0.0001 [R], *p* = 0.0009 [G]) were significantly higher for the sanitizers compared to the non-treated control. However, no significant differences were observed for the B value. 

**Figure 2:**
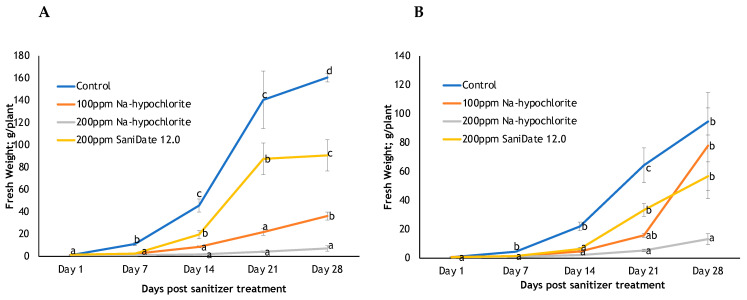
Effect of sanitizers on (**A**) fresh edible lettuce weight (g shoots/plant) and (**B**) fresh edible basil weight (g shoots/plant). Mean values are pooled from three experiments (*n* = 9). Specific time points that share the same letters are not significantly different at the 5% level based on Fisher’s LSD post-hoc analysis. Bars represent the mean standard error.

Basil and lettuce plants grown in the nutrient solution treated with Virkon, KleenGrow, Zerotol, and Green Shield died by Day 5 post-treatment. In nutrient solution treated with bleach (100 ppm, 36.30 ± 3.51 g/plant; 200 ppm 7.17 ± 2.31 g/plant) and SaniDate 12.0 (200 ppm; 90.73 ± 39.50 g/plant), fresh lettuce weight at harvest (Day 28) was significantly lower when compared to the non-treated nutrient solution (160.62 ± 3.88 g/plant; *p* < 0.001) (Figure 2). However, basil fresh weight at harvest was significantly lower when the nutrient solution was treated with 200 ppm bleach (13.22 ± 3.78 g/plant) (*p* = 0.007). The fresh weight of basil on Day 28 for plants from the non-treated system (94.72 ± 9.35 g/plant), 100 ppm bleach (77.97 ± 36.82 g/plant) and 200 ppm SaniDate 12.0 (56.78 ± 9.96 g/plant) were not significantly different. 

#### 3.2.3. Nutritional Value 

At harvest, the total amount of chlorophyll (‘a’ and ‘b’) in lettuce was significantly lower for all treatments compared to the non-treated control (*p* = 0.002) (Table 7). Among the sanitizers, total chlorophyll and chlorophyll ‘a’ concentrations in lettuce were least affected by SaniDate 12.0 (200 ppm) treatment. The ratio of chlorophyll ‘a:b’ was not significantly affected by the treatments (*p* = 0.076). Total carotenoid concentrations in lettuce were significantly reduced by the chlorine-based treatments (100 and 200 ppm) compared with the non-treated control (*p* < 0.001), while the SaniDate 12.0 treatment had no effect on total carotenoids. The total chlorophyll: carotenoid ratio was significantly reduced by all the treatments (*p* < 0.001) compared to non-treated control.

Total chlorophyll and chlorophyll ‘a’ were significantly lower in basil grown in the nutrient solution treated with bleach (100 ppm and 200 ppm) (*p* < 0.05). There were no differences in the total chlorophyll, chlorophyll ‘a:b’, total carotenoid concentrations, or total chlorophyll: carotenoid for basil grown in the nutrient solution treated with 200 ppm SaniDate 12.0 in comparison with the non-treated control at harvest (Day 28) (Table 7). Chlorophyll ‘a:b’ was significantly higher for nutrient solution treated with 200 ppm bleach compared to 100 ppm bleach and the non-treated control (*p* = 0.03). 

## 4. Discussion

Chemical sanitizers are commonly used in commercial greenhouses. Although not all growers use them, many rely on sanitizers to disinfect greenhouse surfaces, equipment, and tools as plant disease management tools. Bleach and quaternary ammonia compounds (QACs), are the primary means of surface disinfection in greenhouses. However, chemical sanitizer efficacy against foodborne bacterial pathogens on surfaces that come into direct contact with the crops has not been extensively explored. 

Chlorine-based sanitizers have been routinely the “go-to” choice among growers for surface disinfection due to their ease of use, availability, and cost-effectiveness [26]. In our study, when used at recommended concentrations (up to 200 ppm) and contact times, bleach and NatriClor CD did not eliminate *Salmonella* Typhimurium from NFT growing channels and top covers (referred to as channels and tops from here on out), main drain lines, or reservoirs. In fact, the efficacy of the bleach treatments was similar to using only water. While 500 ppm bleach has been shown to eliminate biofilm-forming bacteria from PVC coupons after 1 h [27], the high concentrations are not recommended in hydroponics. In fact, the routine use of high concentrations of bleach is known to degrade components of pumps and aerators, seals, and tubing, restricting the usefulness of bleach for surface sanitation in commercial NFT hydroponic operations [28,29]; similar was observed in our study. PPA sanitizers are generally less detrimental to surfaces in processing and hydroponic greenhouses, and thus, their use in surface sanitation is increasing. In this study, Zerotol and the high rate of SaniDate 12.0 (200 ppm) eliminated *S.* Typhimurium from the channels, main drain lines, and reservoir surfaces. Although the lower rate of SaniDate 12.0 (100 ppm) significantly reduced *S.* Typhimurium on most of the surfaces, *S.* Typhimurium was not eliminated. The QACs (Green Shield, KleenGrow) and PPMS-based (Virkon) also eliminated *S*. Typhimurium from the surfaces tested. However, these sanitizers are not labeled for food use, and in our study, we show that their use in the system damaged the plant’s health beyond repair. 

The size, color, and nutritional value of leafy greens are important factors for consumers when purchasing vegetables. At the recommended label rates for surface sanitation, all the sanitizers tested lowered or eliminated *S*. Typhimurium but decreased yield at minimum by 25% and chlorophyll and carotenoid levels by approximately 20%. When the nutrient solution was treated with Green Shield, KleenGrow, Virkon, and Zerotol, all the lettuce and basil plants died within 5 days. Howard et al. (2007) [29] also found that Virkon, Chemprocide (same active ingredient as KleenGrow), and Biosentry 904 (QAC) were phytotoxic to greenhouse-grown vegetables when sprayed onto the leaves to simulate an accidental over-spray, but they did not test leafy greens. Although lettuce and basil did not die after exposure to a nutrient solution treated with bleach, the yield was lower than plants not exposed to the bleach-treated nutrient solution. In addition, chlorophyll and carotenoid levels were lower in plants grown in a bleach-treated nutrient solution, reducing the nutritional value of lettuce and basil. The negative impacts of bleach on plant yield and quality were not unexpected, as bleach is a potent oxidizing agent that damages cell membranes and DNA [8]. Bleach residues are also known to interact with organic material, resulting in harmful byproducts, such as trihalomethane (THM) and halo acetic acid (HAA), increasing chemical food safety risks [8]. The basil and lettuce yields and chlorophyll and carotenoid levels were also reduced when grown in the nutrient solution treated with SaniDate 12.0. However, the effect on yield was not as drastic as the higher rate of bleach. Interestingly, basil was more tolerant to SaniDate 12.0 than lettuce, especially when comparing chlorophyll and carotenoid levels. Only a 5% decrease in chlorophyll was observed in basil after treatment. Unlike chlorine-based sanitizers, PPAs do not produce harmful disinfectant byproducts but rather disassociate to produce water and acetic acid [8,30].

In plant health studies, it is common to use machine modeling and monitoring to assess RGB growth and crop nutrition [30,31,32,33]. In our study, we used RGB as a crop quality indicator, but this approach did not allow us to identify differences in color intensity, chroma, and saturation between the sanitizer treatments. However, we were able to replicate the true color of the lettuce and basil crops based on the HEX codes. Although this does not allow us to make inferences about the nutritional quality of the crop, consumers often use color as an indicator of product freshness, ripeness, and nutrient content [34]. Visually, lettuce and basil plants were paler in color when grown in a chlorine-treated nutrient solution, presenting a bleaching effect compared to the plants grown in SaniDate 12.0 treated nutrient solution or non-treated nutrient solution (Figure S1). 

As a mitigation tool, chlorine-based sanitizers prove to be unsuitable. They were not effective in eliminating *S.* Typhimurium, and the negative effect on plant health occurred immediately after exposure. In addition, the short life cycles of lettuce and basil do not allow sufficient time for the plants to recover post-sanitizer treatment, even though free chlorine levels dropped consistently and were not detected by harvest. In contrast, the study results showed that SaniDate 12.0 at 200 ppm as a one-time treatment had a marginal impact on lettuce and basil crop yield, color, and nutritional value and could be a good alternative to chlorine-based sanitizers. 

## 5. Conclusions

The elimination of human pathogens from NFT systems without compromising plant health remains a critical yet challenging task. According to the United States Environmental Protection Agency (U.S.-EPA)—Office of Inspector General, more than 40% of all registered antimicrobial products have not been tested for efficacy, and between 33% and 50% fail depending on the bacterial human pathogen tested [35].

This study provides evidence about the effectiveness of sanitizers commonly used against *S*. Typhimurium on inanimate surfaces in commercial hydroponic crop production. We identified the limitations of chlorine-based sanitizers and showed the effectiveness of SaniDate 12.0 (200 ppm) in eliminating *S.* Typhimurium on NFT surfaces.

To facilitate chemical sanitizers in a closed system with plants, it is essential that the chosen sanitizer eliminates the target pathogen and does not adversely impact plant yield, quality, or nutritional value. We demonstrated that SaniDate 12.0 (200 ppm) can be applied to nutrient solution at-planting with marginal yield, quality, and nutritional losses on lettuce and basil. Further research is now needed to demonstrate the efficacy of SaniDate 12.0 against *S*. Typhimurium and other bacterial human pathogens in the closed commercial hydroponic system during the production of leafy green plants. 

## Figures and Tables

**Table 1:** Chemical sanitizers used to treat the commercial hydroponic surfaces in this study, including the active ingredients, concentrations, and contact time.

Sanitizer Type	Sanitizer Brand	Active Ingredient (%)	Treatment Concentrations	Contact Time (min)
Chlorine-based	Bleach	Sodium hypochlorite (6)	100 ppm	10
	Bleach	Sodium hypochlorite (6)	200 ppm	10
	NatriChlor™ CD	Aqueous chlorine dioxide (0.3)	10 ppm,	10
	NatriChlor™ CD	Aqueous chlorine dioxide (0.3)	50 ppm	10
PPA ^1^	Zerotol™	Hydrogen peroxide (27.1) and Peroxyacetic acid (2.0)	5%	10
	SaniDate™12.0	Hydrogen peroxide (18.5) and Peroxyacetic acid (12.0)	100 ppm	5
	SaniDate™ 12.0	Hydrogen peroxide (18.5) and Peroxyacetic acid (12.0)	200 ppm	5
Quaternary Ammonia Compounds (QAC) ^3^	KleenGrow™	Didecyl dimethyl ammonium chloride (7.5)	2%	10
	Green Shield™	Alkyl dimethyl benzyl ammonium chloride (10.0) and Alkyl dimethyl ethyl benzyl ammonium chloride (10)	5%	10
PPM ^2,3^	Virkon™	Potassium peroxymonosulfate (21.42) and Sodium chloride (1.50)	1%	10

^1^ PPA—Peroxide-peroxyacetic acid. ^2^ PPM—Potassium peroxymonosulfate-NaCl. ^3^ Not registered for use in the food industry.

**Table 2:** Measures and calculation of chlorophyll (chl) and carotenoid (car) content in lettuce and basil leaves.

Nutrients	Concentration Extract (μg/mL)	Concentration Plant Tissue (μg/g Fresh Weight)
Chlorophyll a	12.25 A_663.2_–2.79 A_646.8_	Chl a extract × volume/g tissue
Chlorophyll b	21.50 A_646.8_–5.1 A_663.2_	Chl b extract × volume/g tissue
Chlorophyll a + b	7.5 A_663.2_ + 18.71 A_646.8_	Chl a + b extract × volume/g tissue
Carotenoid	1000 A_470_–1.8 Chl a–85.02 Chl b/198	Carotenoid extract × volume/g tissue

**Table 3:** Mean log and percent reduction of Salmonella Typhimurium (initial 8 log CFU/cm^2^) following application of commonly used chemical sanitizers on different inanimate surfaces of NFT hydroponic systems (Experiment 1).

Sanitizer Group	Treatment	NFT Surfaces for Hydroponic Production of Leafy Greens			
		Reservoir	Top Cover	Growing Channel	Main Drain Line
		Log Reduction (CFU/cm^2^ ± SE) (% Reduction)	Log Reduction (CFU/cm^2^ ± SE) (% Reduction)	Log Reduction (CFU/cm^2^ ± SE) (% Reduction)	Log Reduction (CFU/cm^2^ ± SE) (% Reduction)
Control	Water Treatment	0.45 ± 0.19 b	0.62 ± 0.10 b	1.53 ± 0.27 b	2.36 ± 0.60 b
		(64.000)	(72.624)	(94.735)	(99.559)
Chlorine-based	Bleach 100 ppm	0.68 ± 0.04 b	1.83 ± 0.43 b	0.68 ± 0.08 b	0.86 ± 0.43 b
		(78.562)	(95.187)	(78.749)	(69.342)
	Bleach 200 ppm	1.78 ± 0.32 ab	3.21 ± 0.4 b	3.42 ± 0.19 ab	0.89 ± 0.07 b
		(93.810)	(99.823)	(99.931)	(86.041)
	NatriChlor™ CD 10 ppm	0.57 ± 0.07 b	1.35 ± 0.23 b	1.37 ± 0.51 b	1.21 ± 0.21 b
		(70.781)	(91.739%)	(90.616%)	(90.979%)
	NatriChlor™ CD 50 ppm	0.67 ± 0.10 b	4.14 ± 0.40 ab	2.39 ± 0.29 b	2.46 ± 0.06 b
		(73.500)	(99.907)	(98.763)	(99.643)
PPA	Zerotol™ 5%	* 5.60 ± 0.00 a	* 5.18 ± 0.00 a	* 5.15 ± 0.00 a	* 5.21 ± 0.00 a
	SaniDate™ 12.0 100 ppm	* 5.60 ± 0.00 a	* 5.18 ± 0.00 a	4.49 ± 0.35 a	4.24 ± 0.51 a
				(99.971)	(99.926)
	SaniDate™ 12.0 200 ppm	* 5.60 ± 0.00 a	* 5.18 ± 0.00 a	* 5.15 ± 0.00 a	* 5.21 ± 0.00 a
PPM	Virkon™ 1%	* 5.60 ± 0.00 a	5.18 ± 0.00 a	5.15 ± 0.00 a	* 5.21 ± 0.00 a
Quaternary ammonia	KleenGrow™ 2%	* 5.60 ± 0.00 a	* 5.18 ± 0.00 a	* 5.15 ± 0.00 a	* 5.21 ± 0.00 a
	Green Shield™ 5%	* 5.60 ± 0.00 a	* 5.18 ± 0.00 a	* 5.15 ± 0.00 a	* 5.21 ± 0.00 a
	*p*-value	<0.001	<0.001	<0.001	<0.001

Each data point represents the mean log reduction and standard error derived from four replications with two samples per replication (*n* = 8) in experiment 1. Values in parenthesis represent the mean percentage reduction. Asterisk (*) indicates a 100% reduction and no detection after enrichment. Values in a column followed by different letters are significantly different at *p* < 0.05 based on Fisher’s LSD post-hoc analysis.

**Table 4:** Mean log and percent reduction of Salmonella Typhimurium (initial 8 log CFU/cm^2^) following application of commonly used chemical sanitizers on different inanimate surfaces of NFT hydroponic systems (Experiment 2).

Sanitizer Group	Treatment	NFT Surfaces for Hydroponic Production of Leafy Greens			
		Reservoir	Top Cover	Growing Channel	Main Drain Line
		Log Reduction (CFU/cm^2^ ± SE) (% Reduction)	Log Reduction (CFU/cm^2^ ± SE) (% Reduction)	Log Reduction (CFU/cm^2^ ± SE) (% Reduction)	Log Reduction (CFU/cm^2^ ± SE) (% Reduction)
Control	Water Treatment	4.49 ± 0.07 b	4.28 ± 0.34 b	3.68 ± 0.35 b	4.90 ± 0.16 b
		(99.997)	(99.990)	(99.951)	(99.997)
Chlorine-based	Bleach 100 ppm	3.90 ± 1.18 b	3.65 ± 0.61 b	4.22 ± 0.54 bc	4.98 ± 0.14 ab
		(99.904)	(99.870)	(99.970)	(99.998)
	Bleach 200 ppm	4.61 ± 0.33 b	4.67 ± 0.30 ab	☩ 5.07 ± 0.08 ac	☩ 5.20 ab
		(99.996)	(99.996)	(99.999)	(100.000)
	NatriChlor™ CD 10 ppm	☩ 5.08 ab	3.90 ± 0.32 b	4.17 ± 0.28 b	4.87 ± 0.23 ab
		(99.999)	(99.977)	(99.986)	(99.998)
	NatriChlor™ CD 50 ppm	☩ 5.34 ± 0.26 ab	4.13 ± 0.03 b	4.83 ± 0.23 bc	* 5.11 ± 0.09 ab
		(99.999)	(99.991)	(99.997)	(99.999)
PPA	Zerotol™ 5%	* 5.60 ± 0.00 a	* 5.18 ± 0.00 a	* 5.15 ± 0.00 a	* 5.20 ab
	SaniDate™ 12.0 100 ppm	* 5.60 ± 0.00 a	* 5.18 ± 0.00 a	* 5.15 ± 0.00 a	5.03 ± 0.10 ab
	SaniDate™ 12.0 200 ppm	* 5.60 ± 0.00 a	* 5.18 ± 0.00 a	* 5.15 ± 0.00 a	* 5.20 ± 0.00 a
PPM	Virkon™ 1%	* 5.60 ± 0.00 a	5.18 ± 0.00 a	* 5.15 ± 0.00 a	* 5.20 ± 0.00 a
Quaternary ammonia	KleenGrow™ 2%	* 5.60 ± 0.00 a	* 5.18 ± 0.00 a	* 5.15 ± 0.00 a	* 5.20 ± 0.00 a
	Green Shield™ 5%	* 5.60 ± 0.00 a	* 5.18 ± 0.00 a	* 5.15 ± 0.00 a	* 5.20 ± 0.00 a
	*p*-value	0.039	<0.001	<0.001	0.097

Each data point represents the mean log reduction and standard error derived from four replications with two samples per replication (*n* = 8) in experiment 2. Values in parenthesis represent the mean percentage reduction. Plus (☩) indicates recovery after enrichment for sanitizer treatment with 99.999–100% reduction. Asterisk (*) indicates 100%reduction and no detection after enrichment. Values in a column followed by different letters are significantly different at *p* < 0.05 based on Fisher’s LSD post-hoc analysis.

**Table 5:** Free chlorine, total chlorine, oxidative reduction potential (ORP) of the nutrient solution following treatment with bleach.

Bleach	Day	Free Chlorine ^1^ (mg/L)	Total Chlorine ^1^ (mg/L)	ORP (mV)	Relative ORP (∆ORP) (mV)	Temperature of Nutrient Solution (°C)
100 ppm	1	7.49 ± 3.55	25.73 ± 11.87	872.33 ± 33.45	203.00 ± 33.5	22.64 ± 1.32
	2	3.67 ± 0.68	9.90 ± 1.87	775.75 ± 55.78	113.50 ± 34.65	11.23 ± 2.24
	7	0.15 ± 0.29	<1.0	417.93 ± 152.16	38.63 ± 39.68	22.53 ± 1.68
	14	<0.10	<1.0	448.20 ± 65.99	^2^ 17 ± 6.63	23.86 ± 2.30
200 ppm						
	1	>25	110.69 ± 31.09	857.67 ± 25.45	194.50 ± 30.97	23.35 ± 1.70
	2	18.63 ± 4.32	69.90 ± 9.84	830.25 ± 28.34	144.00 ± 36.77	19.78 ± 2.59
	7	6.71 ± 6.25	16.46 ± 13.40	788.79 ± 1.58	365.38 ± 134.59	23.89 ± 2.71
	14	0.81 ± 1.69	1.25 ± 1.81	534.10 ± 200.14	33.80 ± 71.69	23.78 ± 2.04

^1^ Free and total chlorine concentrations were determined with the CHR (high concentration of chlorine) sensor (Palin Kemio, England). The detection range for free chlorine was 0.1–25 mg/L (5–25 °C) and 1–500 mg/L (5–30 °C) for total chlorine. ^2^ Only values higher than the control were used to calculate relative ORP. Each data point represents the mean and standard deviation.

**Table 6:** The color of lettuce and basil grown in sanitizer-treated nutrient solution at harvest expressed as red-green-blue (RGB) values and the hexadecimal color (HEX) code.

		R	G	B	HEX Code
Lettuce	Non-treated (control)	139.33 ± 24.25	195.33 ± 12.19	52.33 ± 10.61	#8BC334
	Bleach 100 ppm	136.67 ± 11.17	190.17 ± 8.32	45.67 ± 3.95	#BDE254
	Bleach 200 ppm	138.50 ± 16.46	189.83 ± 11.93	28.50 ± 12.88	#8BBE1D
	SaniDate 12.0 200 ppm	127.38 ± 6.76	192.88 ± 7.76	39.50 ± 8.73	#7FC128
Basil	Non-treated (control)	114.17 ± 13.10	177.50 ± 15.73	75.83 ± 12.24	#72B24C
	Bleach 100 ppm	189.00 ± 17.69	226.33 ± 6.58	84.00 ± 6.82	#BDE254
	Bleach 200 pm	173.67 ± 9.33	220.17 ± 8.93	69.33 ± 5.50	#AEDC45
	SaniDate 12.0 200 ppm	135.88 ± 5.40	199.88 ± 5.35	86.38 ± 6.64	#88C856

Each RGB color represents the mean values and standard error pooled from three experiments (*n* = 9).

**Table 7:** Effect of chemical sanitizers commonly used in commercial greenhouses on the nutritional value of lettuce and basil grown in a nutrient film technique (NFT) hydroponic system.

		Nutritional Value					
		Chlorophyll (μg/g. FW)				Carotenoid (μg/g. FW)	Total Chlorophyll: Carotenoid
		Total	“a”	“b”	“a:b”		
Lettuce	Non-treated	2004.30 ± 104.27 A	1206.05 ± 54.53 A	453.64 ± 19.78 A	2.66 ± 0.02 A	321.29 ± 21.78 A	6.27 ± 0.12 A
	Bleach 100 ppm	1100.79 ± 99.03 B	673.96 ± 64.17 B	250.45 ± 24.50 B	2.70 ± 0.05 A	232.11 ± 14.19 B	4.72 ± 0.20 B
	Bleach 200 ppm	730.75 ± 73.64 C	430.72 ± 48.80 C	147.07 ± 21.33 C	3.01 ± 0.13 A	116.82 ± 13.29 C	4.36 ± 0.19 C
	SaniDate 12.0 200 ppm	1650.95 ± 109.30 D	1051.67 ± 61.33 A	352.25 ± 24.12 D	3.04 ± 0.21 A	280.53 ± 14.35 A	5.85 ± 0.12 C
	*p*-value	<0.001	<0.001	<0.001	0.076	<0.001	<0.001
Basil	Non-treated	1105.11 ± 77.16 A	661.35 ± 43.29 A	235.40 ± 17.07 A	2.82 ± 0.05 A	235.73 ± 18.09 A	4.71 ± 0.07 A
	Bleach 100 ppm	727.31 ± 29.47 B	430.16 ± 18.34 B	146.51 ± 8.14 A	2.95 ± 0.06 A	186.91 ± 6.51 A	3.90 ± 0.12 A
	Bleach 200 ppm	490.74 ± 22.11 B	282.74 ± 18.08 B	78.24 ± 7.06 A	3.66 ± 0.11 B	149.85 ± 3.09 A	3.27 ± 0.12 A
	SaniDate 12.0 200 ppm	1047.37 ± 162.36 A	738.94 ± 159.95 A	357.67 ± 191.02 A	3.23 ± 0.35 AB	110.89 ± 89.54 A	3.44 ± 1.08 A
	*p*-value	0.002	0.018	0.276	0.03	0.426	0.39

Each data point represents the combined mean (*n* = 9) and standard error of three independent experiments. Means within the same column followed by the same letters are not significantly different at the 5% level based on Fisher’s LSD post-hoc analysis.

## Data Availability

The original contributions presented in the study are included in the article/Supplementary Materials, further inquiries can be directed to the corresponding authors. The raw data supporting the conclusions of this article will be made available by the authors on request.

## Supplementary Materials

The following supporting information can be downloaded at: https://www.mdpi.com/article/10.3390/foods13121929/s1, Figure S1: RGB color and visual appearance of lettuce and basil plants when grown in the sanitizer-treated nutrient solution. (A) Lettuce RGB color changes during the crop cycle and the visual appearance of lettuce leaves at harvest (Day 28). (B) Basil RGB color changes during the crop cycle and the visual appearance of lettuce leaves at harvest (Day 28). The images were pictures taken at harvest.
