# Supplementary material for: Effectiveness of Chemical Sanitizers against Salmonella Typhimurium in Nutrient Film Technique (NFT) Hydroponic Systems: Implications for Food Safety, Crop Quality, and Nutrient Content in Leafy Greens
Source: Foods. 2024 Jun 19;13(12):1929. doi: 10.3390/foods13121929 (PMC11203249; doi:10.3390/foods13121929)
Supplement: Supplementary file 1 [file foods-13-01929-s001.zip › foods-3036995-supplementary.pdf]

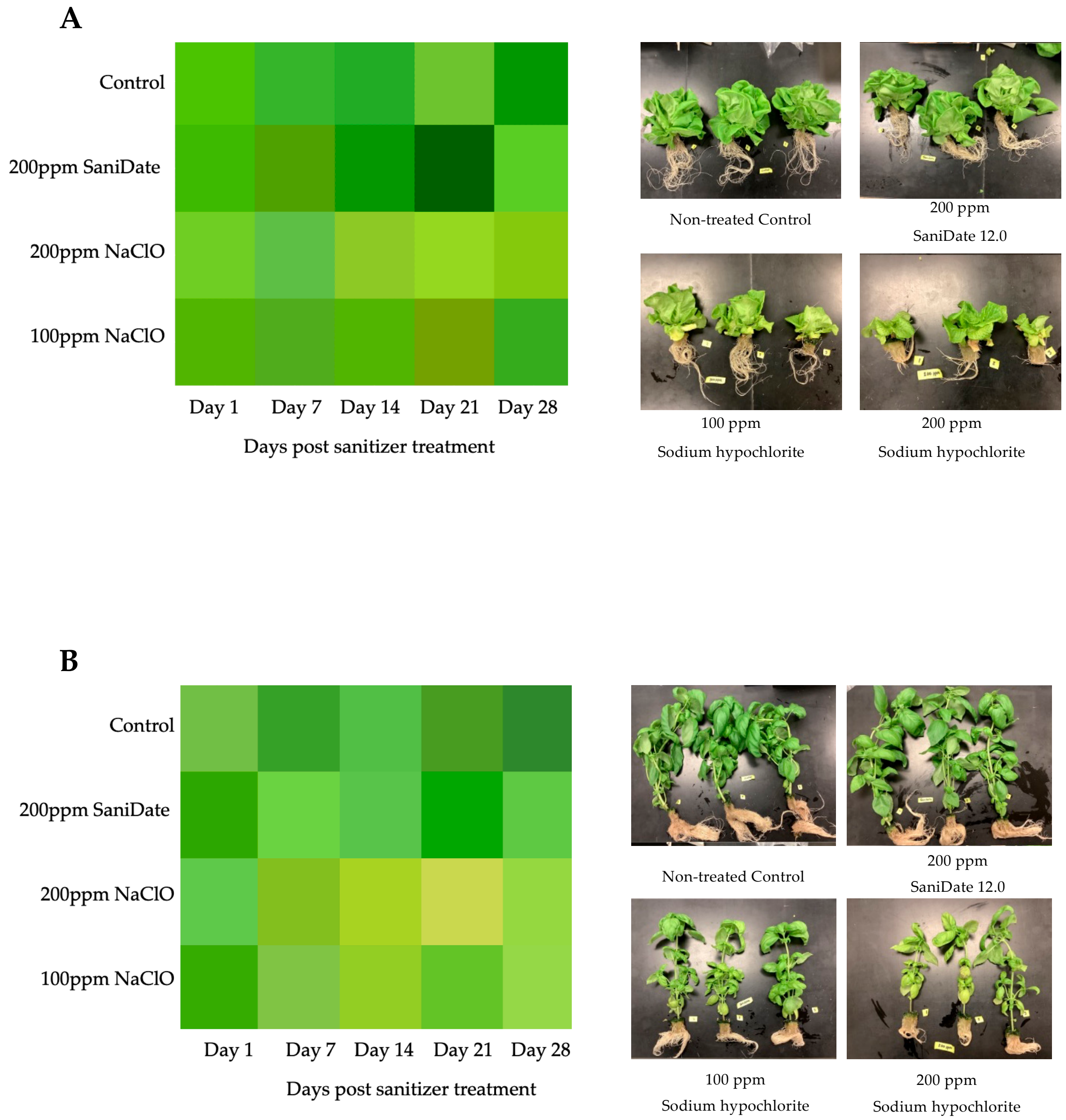

Figure S1: RGB color and visual appearance of lettuce and basil plants when grown in sanitizer-treated nutrient solution. (A) Lettuce RGB color changes during the crop cycle and the visual appearance of lettuce leaves at harvest (Day 28). (B) (B) Basil RGB color changes during the crop cycle and the visual appearance of lettuce leaves at harvest (Day 28). The images were pictures taken at harvest.
